# Cost comparison of nine-month treatment regimens with 20-month standardized care for the treatment of rifampicin-resistant/multi-drug resistant tuberculosis in Nigeria

**DOI:** 10.1371/journal.pone.0241065

**Published:** 2020-12-01

**Authors:** Florence O. Bada, Nick Blok, Evaezi Okpokoro, Saswata Dutt, Christopher Akolo, Patrick Dakum, Alash’le Abimiku

**Affiliations:** 1 International Research Center of Excellence, Institute of Human Virology Nigeria, Abuja, Nigeria; 2 Department of Epidemiology and Public Health, University of Maryland Graduate School, Baltimore, Maryland, United States of America; 3 KNCV Tuberculosis Foundation, The Hague, The Netherlands; 4 FHI 360, Washington, DC, United States of America; 5 Department of Prevention, Care and Treatment, Institute of Human Virology, Abuja, Nigeria; 6 University of Maryland School of Medicine, Baltimore, Maryland, United States of America; Harvard Medical School, UNITED STATES

## Abstract

**Background:**

Globally, drug resistant tuberculosis (DR-TB) continues to be a public health threat. Nigeria, which accounts for a significant proportion of the global burden of rifampicin/multi-drug resistant-TB (RR/MDR-TB) had a funding gap of $168 million dollars for TB treatment in 2018. Since 2010, Nigeria has utilized five different models of care for RR/MDR-TB (Models A-E); Models A, B and C based on a standardized WHO-approved treatment regimen of 20–24 months, were phased out between 2015 and 2019 and replaced by Models D and E. Model D is a fully ambulatory model of 9–12 months during which a shorter treatment regimen including a second-line injectable agent is utilized. Model E is identical to Model D but has patients hospitalized for the first four months of care while Model F which is to be introduced in 2020, is a fully ambulatory, oral bedaquiline-containing shorter treatment regimen of 9–12 months. Treatment models for RR/MDR-TB of 20–24 months duration have had treatment success rates of 52–66% while shorter treatment regimens have reported success rates of 85% and above. In addition, replacing the second-line injectable agent in a shorter treatment regimen with bedaquiline has been found to further improve treatment success in patients with fluoroquinolone-susceptible RR/MDR-TB.

Reliable cost data for RR/MDR-TB care are limited, specifically costs of models that utilize shorter treatment regimens and which are vital to guide Nigeria through the provision of RR/MDR-TB care at scale. We therefore conducted a cost analysis of shorter treatment regimens in use and to be used in Nigeria (Models D, E and F) and compared them to three models of longer duration utilized previously in Nigeria (Models A, B and C) to identify any changes in cost from transitioning from Models A-C to Models D-F and opportunities for cost savings.

**Methods:**

We obtained costs for TB diagnostic and monitoring tests, in-patient and out-patient care from a previous study, inflated these costs to 2019 NGN and then converted to 2020 USD. We obtained other costs from the average of six health facilities and drug costs from the global drug facility. We modeled treatment on strict adherence to two Nigerian National guidelines for programmatic and clinical management of drug-resistant tuberculosis.

**Results:**

We estimated that the total costs of care from the health sector perspective for Models D, E and F were $4,334, $7,705 and $3,420 respectively. This is significantly lower than the costs of Models A, B and C which were $14,781, $12, 113, $7,572 respectively.

**Conclusion:**

Replacing Models A–C with Models D and E reduced the costs of RR/MDR-TB care in Nigeria by approximately $5,470 (48%) per patient treated and transitioning from Models D and E to Model F would result in further cost savings of $914 to $4,285 (21 to 56%) for every patient placed on Model F. If the improved outcomes of patients managed using bedaquiline-containing shorter treatment regimens in other countries can be attained in Nigeria, Model F would be the recommended model for the scale up of RR/MDR-TB care in Nigeria.

## Introduction

Globally, drug resistant tuberculosis (DR-TB) continues to be a public health threat. In 2018, there were approximately half a million new cases of rifampicin resistant tuberculosis (RR-TB) of which 78% had multi-drug resistant tuberculosis (MDR-TB) [1]. MDR-TB is mycobacterium tuberculosis with simultaneous resistance to at least isoniazid and rifampicin. Once the presence of RR/MDR-TB is established, treating tuberculosis effectively requires the use of a combination of complex and sometimes more costly second-line drugs for a prolonged period of time [2–4].

Programmatic Management of drug-resistant TB (PMDT) was introduced as a specific approach for managing MDR-TB globally in 1999 [5]. While this initially involved hospitalization for a period of 20–24 months, it has since evolved to treatment models with reduced hospitalization and currently includes fully-ambulatory models. These initial models of 20–24 months duration ranged in cost from $1,839 to $81,000 per patient treated [4,6–9] and had success rates between 52–66% [10–14]. In 2016, the World Health Organization (WHO) recommended the utilization of Shorter Treatment Regimen [15] based on reported success rates of 85% and above [16–19]. These shorter treatment regimen models have been implemented in several countries at costs ranging between $4,552 and $6,618 [20]. WHO now recommends a further modification; the use of a shorter, all oral, bedaquiline-containing regimen for eligible patients: patients without previous exposure to second-line medicines for more than one month, without fluoroquinolone resistance and in the absence of extensive TB disease or severe extra-pulmonary TB [21]. This shift away from regimen containing injectable second-line anti-TB drugs (kanamycin, amikacin or capreomycin) is due to the myriad of challenges associated with their use; logistical and psychological challenges of daily injections as well as adverse drug reactions (ADRs) most notably irreversible ototoxicity and acute renal injury [22–24]. Replacing the injectable agent in a shorter treatment regimen with bedaquiline has the added advantage of improving treatment success in patients with fluoroquinolone-susceptible RR/MDR-TB [25,26].

Nigeria, which as of 2018 had a projected 21,000 cases of drug resistant tuberculosis, is one of ten countries responsible for 75% of the gap between DR-TB cases notified and treatment enrollments [1]. Nigeria commenced PMDT in 2010 with the WHO-recommended 20–24 month standardized regimen which was implemented through hospitalization of patients at specialized treatment centers and has transitioned through several models with varied lengths of treatment in the ensuing ten years. Models used in Nigeria to address RR/MDR-TB (Models A-E) are described in “Table 1”. Nigeria is expected to introduce the fully oral bedaquiline-containing shorter treatment regimen for eligible patients in 2020 (Model F in “Table 1”).

**Table 1:** Models of care for drug-resistant tuberculosis patients in Nigeria (2010–2020).

Model of Care	Drug Regimen	Regimen Combination	Duration of Treatment	Duration of Intensive Phase	Length of Hospitalization	Status
A	Standardized Care (8 Km-Lfx-Pto-Cs-Z/12 Lfx Pto-Cs-Z)	Injectable and oral	20 months	8 months	8 months	Phased Out in 2015
B	Standardized Care (8 Km-Lfx-Pto-Cs-Z/12 Lfx-Pto-Cs-Z)	Injectable and oral	20 months	8 months	5 months	Phased out from 2017–2019
C	Standardized Care (8 Km-Lfx-Pto-Cs-Z/12 Lfx-Pto-Cs-Z)	Injectable and oral	20 months	8 months	No hospitalization	Phased out in 2019
D	Standardized Care (4–6 Am-Mfx-Cfz-Pto-Z-E-H^h^/5 Mfx-Cfz-E-Z)	Injectable and oral	9–12 months	4–6 months	No Hospitalization	To be phased out in 2021
E	Standardized Care (4–6 Am-Mfx-Cfz-Pto-Z-E-H^h^/5 Mfx-Cfz-E-Z)	Injectable and oral	9–12 months	4–6 months	4 months	To be phased out in 2021
F	Standardized Care (4–6 Bdq(6m)-Mfx-Cfz- -Z-E- H^h^-Pto/5-Mfx-Cfz-Z-E)	Fully Oral	9–12 months	4–6 months	No Hospitalization	To be rolled out in 2020

List of abbreviations: Km = kanamycin, Lfx = levofloxacin, Pto = prothionamide, Cs = cycloserine, Z = pyrazinamide, Am = amikacin, Mfx = moxifloxacin, Cfz = clofazimine, E = ethambutol, Bdq = bedaquiline.

In 2018, Nigeria, had a funding gap of $168 million dollars for TB treatment and care [1].Therefore, careful planning and judicious use of available funds are required to guide the country as it scales up RR/MDR-TB care. However, cost data for RR/MDR-TB treatment are limited [27], specifically costs of models that utilize shorter treatment regimen to manage RR/MDR-TB. Though the costs of Models A, B and C previously used in Nigeria have been published [7], the costs of Models D, E and F are not known. We therefore conducted a cost-analysis of the shorter treatment regimen in use (Models D and E) as well as one planned to be used to treat RR/MDR-TB in Nigeria (Models F) and compared them to the three models of longer duration previously used in Nigeria though discontinued between 2015 and 2017 (Models A, B and C) “Table 1”. This analysis has the advantage of comparing six models used within the same geo-political region and was geared towards identifying any changes in cost from transitioning from models A-C to models D-F and potential opportunities for cost savings.

## Materials and methods

### Setting

Persons identified as being at risk of DR-TB infection are first tested with Xpert MTB/RIF and those confirmed to have RR-TB are further tested with first- and second-line line probe assay (LPA), culture and phenotypic DST. Patients with RR/MDR-TB in Nigeria are then managed using PMDT “Table 1”. Models A, B and C were based on a standardized WHO-approved treatment regimen of 20–24 months adapted to be implemented over 20 months and which consisted of an eight month intensive phase during which patients received pyrazinamide (Z) and four second-line anti-TB drugs namely levofloxacin (Lfx), kanamycin (Km) (replaced by capreomycin (Cm) when indicated), prothionamide (Pto) and cycloserine (Cs) and a continuation phase of 12 months during which Km was discontinued. Models A, B and C differed only in the duration of hospitalization “Table 1”.

Models D and E were introduced in 2017; Model D is a fully ambulatory model in which a shorter treatment regimen of 9–12 months is utilized. The Intensive Phase consists of 4–6 months during which seven anti-TB drugs are utilized namely amikacin (Am), moxifloxacin (Mfx), clofazimine (Cfz), Pto, Z, ethambutol (E) and high dose isoniazid (H^h^) while the continuation phase lasts for five months during which patients receive Mfx, Cfz, Pto, Z, E and H^h^. Model E is the same duration as Model D with identical anti-TB drugs but has patients hospitalized for the first four months of treatment. Model F which is in line with WHO’s consolidated guidelines on tuberculosis (2020) [21] is to be introduced in Nigeria in 2020 and is a fully ambulatory, fully oral bedaquiline-containing shorter treatment regimen of 9–12 months duration with a 4–6 month intensive phase during which patients receive six months of bedaquiline (Bdq) which could stretch into the continuation phase, and 4–6 months of Mfx, Cfz, Pto, Z, E and H^h^. Model F has a continuation phase of five months during which patients receive Mfx, Cfz, Pto, Z, E.

DR-TB care in Nigeria is coordinated by a DR-TB Technical Committee at the National Level and by a DR-TB Management Team at State Level. There is close patient monitoring with daily home visits by the direct observation of treatment (DOT) officer during the intensive phase for patients on injectable agents which becomes bi-monthly home visits during the continuation phase, interspersed by bi-monthly visits of patients to the DOT clinics for monitoring and drug pick-ups. There are also monthly home visits by the TB and leprosy supervisor overseeing the local government in which the patient lives, monthly home visits by the State DR-TB focal person and quarterly home visits by the State consilium which is a multi-disciplinary Team [7]. A full description of the nature, frequency and timing of out-patient consultations and supervision including home visits is provided below “Table 2”.

**Table 2:** Description of frequency of outpatient consultations and supervision via home-visits for RR/MDR-TB patients managed using Models D-F in Nigeria.

		Model D		Model E		Model F	
Frequency	Unit	Intensive phase	Continuation phase	Intensive phase	Continuation phase	Intensive phase	Continuation phase
Consultation at treatment center/Monthly clinic visit	Visit	5	5	0	5	5	5
Visits to collect medication at DOT center/DOT at DOT center	Visit	8	10	0	10	8	10
Home visit—by DOT officer	Home visit	120	10	0	10	8	10
Home visit by DR-TB focal person	Home visit	4	5	0	5	4	5
Home visit by TBL supervisor	Home visit	4	5	0	5	4	5
Quarterly state team meeting	Meeting	2	1	0	1	2	1
Quarterly state team home visit	Home visit	2	1	0	1	2	1

List of abbreviations: DOT = direct observation of treatment, DR-TB = drug resistant tuberculosis, TBL = tuberculosis and leprosy.

### Costing of Models D-F

We performed a cost analysis to better understand the costs and important drivers of cost across three models of RR/MDR-TB care in Nigeria (Models D—F). The cost analysis was from the perspective of the Nigerian NTP (health sector perspective) and included all DR-TB related management costs such as diagnostic and monitoring tests, clinic visits, home visits, treatment supervision and drugs. We assumed strict adherence to “Guidelines on the use of shorter treatment regimen and new drugs in the clinical and programmatic management of drug-resistant tuberculosis and co-infections in Nigeria (2017)”; an addendum to the NTBLCP 2016 PMDT guideline for models D-F. The time horizon was 9 months for Models D-F based on the Nigerian Program. Ethical approval was obtained from the Nigerian National Health Research Ethics Committee under number NHREC/01/01/2007-29/09/2015b. No consent from individual patients was required for this study. The study entailed direct observation of health care workers as they cared for MDR-TB patients as well as interviewing health care workers to obtain the required information to calculate the health service costs of providing care to MDR-TB patients. Written informed consent was obtained from health care workers prior to interviewing them.

### Unit costs

#### Diagnostic and monitoring tests

We obtained the costs of TB diagnostic and monitoring tests “Table 3” such as smear microscopy, Xpert MTB/RIF, culture (liquid and solid), line probe assay (LPA) and drug susceptibility testing (DST) from a previous study on Models A, B and C in Nigeria [7]. These costs were obtained in 2014 NGN, inflated to 2019 NGN using the annual inflation of consumer prices in the period 2014–2019 [28] and then expressed in USD using the exchange rate of April 3rd 2020 of 1 USD = 367 NGN [29]. Total inflation for the period 2014–2019 in Nigeria was 83.5%. The exchange rate of the USD versus the naira went from $1 = 158 NGN in 2014 to $1 = 367 NGN in 2020; an appreciation of the USD of 132%. As a result, the costs of TB specific tests obtained in 2014 inflated with consumer prices from 2014–2019 and expressed in USD using the exchange rate of April 3^rd^, 2020 decreased by 21% mainly due to the appreciation of the USD versus the naira.

**Table 3:** Cost of tuberculosis-specific diagnostic and monitoring tests in USD.

Tuberculosis-specific tests	Cost per unit (USD 2014)	Cost per unit (USD 2020)
Gene Xpert	24.64	19.46
Sputum smear	6.34	5.00
Sputum culture–Liquid	97.20	76.78
First and second-line DST	68.23	53.89
First and second-line LPA	76.02	60.05

List of abbreviations: DST = drug susceptibility testing, LPA = line-probe assay.

We obtained the costs of ancillary monitoring tests from the average of six different health facilities “Table 4”.

**Table 4:** Cost of ancillary monitoring tests in USD.

Ancillary monitoring tests	Cost per unit (USD 2020)
Chest X-ray	5.59
Audiometry	29.06
Visual Acuity	13.62
Full blood count	5.50
Thyroid function test	35.42
Liver function test	12.06
Blood Glucose (Fasting)	2.45
HIV test	3.09
CD4	26.34
HIV Viral load	88.56
Pregnancy test	2.59
Electrocardiograph	7.95
Hepatitis B & C	7.87
Serum amylase	6.81

List of abbreviations: CD4 = Cluster of differentiation 4.

#### Drug costs

The drug costs “Table 5” were based on the Global Drug Facility price list which is the sole source for all the drugs used for RR/MDR-TB management in Nigeria.

**Table 5:** Costs of anti-TB drugs in USD.

Drug	Cost (USD) per formulation
Pyrazinamide 400 mg)	0.02 per tablet
Bedaquiline 100mg	2.13 per tablet
Moxifloxacin 400 mg	0.25 per caplet
Levofloxacin 500 mg	0.05 per tablet
Prothionamide 250 mg	0.12 per tablet
High dose INH 300mg	0.02 per capsule
Clofazimine 100mg	0.90 per capsule
Ethambutol 400mg	0.04 per tablet
Cycloserine 250mg	0.25 per capsule
Amikacin 500mg	0.68 per vial
Pyridoxine 100mg	0.05 per tablet
Kanamycin 1g	0.19 per vial

### Cost of in-patient stay and out-patient care

Costs for one bed day, out-patient consultations, home visits and meetings “Table 6” were based on data we had collected previously from six health facilities: three treatment centers providing hospitalization as part of care and three DOTS centers providing ambulatory DR-TB care. These costs were built using an ingredients or bottom-up approach described in great detail in a previous publication [7]. However, in brief, in-patient costs included capital costs for equipment, vehicles and furniture, personnel costs, other recurring costs such as utilities and maintenance and ancillary costs including catering and laundry services [7]. Shared costs including salaries, furniture, supervision, transportation and vehicles were estimated through observation and interview with staff [7]. Costs of outpatient consultations and supervision consisted of salary costs, over-head costs, transportation and other costs. Other costs included the cost of protective gear and supplies for giving a patient an injectable agent and varied based on the purpose of the visit and the number of staff present for each visit. The supplies associated with giving a patient an injectable agent were only included for home visits by DOTS officers in the intensive phase. We inflated these costs from 2014 NGN to 2019 NGN and then converted to 2020 USD.

**Table 6:** Costs of in-patient and out-patient care in USD.

Type of consultation/visit	Cost (2014)	Cost (2020)
Inpatient hospitalization (one bed day)	53.19	42.01
Consultation at Treatment Center/Monthly clinic visit	16.21	12.80
Visit to collect medication at DOT Center/DOT at DOT Center	4.45	3.52
Home visit by DOT Officer	11.21	8.79
Home visit by DR-TB focal person	32.32	25.53
Home visit by TBL supervisor	10.35	8.18
Quarterly State Team Meeting	59.83	47.26
Quarterly State Team home visit	186.13	147.02

### Frequencies

Frequencies of drugs used, tests conducted, and visits for Models D, E and F were derived from the National Guidelines on the use of shorter treatment regimen and new drugs in the clinical and programmatic management of drug-resistant tuberculosis and co-infections in Nigeria 2017 and are provided in “Table 7” below. For our analysis, we assumed strict adherence to this guideline.

**Table 7:** Frequencies of laboratory tests and patient monitoring visits per patient and model for Models D, E and F according to the Nigerian DR-TB guideline in 2017.

		Model D		Model E		Model F	
Frequency	Unit	Intensive phase	Continuation phase	Intensive phase	Continuation phase	Intensive phase	Continuation phase
**I. DR-TB Diagnostic tests**							
Gene Xpert	Test	1	0	1	0	1	0
Sputum smear	Smear	1	0	1	0	1	0
Culture–Liquid	Test	1	0	1	0	1	0
FL LPA	test	1	0	1	0	1	0
SL LPA	Test	1	0	1	0	1	0
1st and 2nd line DST—Solid culture	Test	1	0	1	0	1	0
X-ray	Test	1	0	1	0	1	0
Audiometry test	Test	1	0	1	0	0	0
Visual Acuity	Test	1	0	1	0	1	0
FBC	Test	1	0	1	0	1	0
E, U, Cr	Test	1	0	1	0	1	0
Thyroid function test	Test	1	0	1	0	1	0
Liver Function Test	Test	1	0	1	0	1	0
Blood Glucose (Fasting)	Test	1	0	1	0	1	0
HIV Test	Test	1	0	1	0	1	0
CD4*	Test	0.12	0	0.12	0	0.12	0
Viral Load*	Test	0.12	0	0.12	0	0.12	0
Pregnancy test	Test	0.5	0	0.5	0	0.5	0
ECG	Test	1	0	1	0	1	0
Hepatitis B & C	Test	1	0	1	0	1	0
Serum amylase & lipase	Test	0	0	0	0	1	0
**II. DR-TB monitoring test**							
X-ray	Test	0	2	0	2	0	2
Audiometry test	Test	4	0	4	0	0	0
E, U, Cr	Test	4	0	4	0	4	0
Thyroid function test	Test	1	0	1	0	1	0
LFT	Test	2	1	2	1	2	1
CD4*	Test	0	0.12	0	0.12	0	0.12
Viral Load*	Test	0	0.12	0	0.12	0	0.12
ECG	Test	5	5	5	5	5	5
**III. Medication**							
Pyrazinamide 400 mg	Tablet	600	750	600	750	0	0
Bedaquiline 100mg	Tablet	0	0	0	0	188	0
Amikacin 500mg	Vial	240	0	240	0	0	0
Moxifloxacin 400 mg	Caplet	240	300	240	300	240	300
Levofloxacin 500 mg	Tablet	0	0	0	0	0	0
Prothionamide 250 mg	Tablet	480	0	480	0	480	0
High dose INH 300mg	Capsule	240	0	240	0	240	0
Pyridoxine 100 mg	Tablet	120	150	120	150	120	150
Clofazimine 100mg	Capsule	120	150	120	150	120	150
Ethambutol 400mg	Tablet	360	450	360	450	360	450
**IV. Inpatient stay**							
Inpatient hospitalization days	Bed day	0	0	120	0	0	0
**VI. Follow-up DR-TB testing**							
Sputum smear	Smear	4	5	4	5	4	5
Culture—Liquid	Test	4	5	4	5	4	5

List of abbreviations: Cr = creatinine, DR = drug resistant, DST = drug susceptibility testing, E = electrolytes, ECG = electrocardiograph, ENT = ear nose and throat, FBC = full blood count, FL LPA = first-line line probe assay, SL LPA = second-line line probe assay, TB = tuberculosis, U = urea.

*Based on HIV prevalence in incident TB cases in Nigeria [1].

### Costing of Models A-C

Though the costs of Models A–C had been published previously [7], we recalculated the costs using the same methods described above for Models D-F to account for inflation from 2014 to 2019 and appreciation of the USD versus the naira. Frequencies of drugs used, tests conducted, and visits were derived from the Nigerian National guideline for programmatic and clinical management of drug-resistant tuberculosis in Nigeria (2016) for Models A, B and C and published previously [7] but are included in the supplementary tables “S1 Table”. The time horizon was 20 months for Models A-C based on the Nigerian Program.

## Results

### The costs of nine-month treatment regimen (Models D, E and F)

The total cost of care in USD from the health sector perspective for models D, E and F were $4,334 per patient for Model D, $7,705 per patient for Model E and $3,420 per patient for Model F “Table 8”. The costs of baseline tests, follow-up tests and drugs were identical for Models D and E. In-patient costs for Model E resulted in Model E being almost twice the cost of Model D even with increased costs for out-patient care for a fully ambulatory model. Model F differed from Models D and E in all cost categories: baseline tests and follow-up tests were decreased for Model F while drug costs for Model F were higher.

**Table 8:** The total cost of Models D, E and F in USD.

	Model D	Model E	Model F
Total baseline	364.46	364.46	342.20
Total follow up	1,074.60	1,074.60	958.35
Total drugs	674.39	674.39	883.28
Total inpatient stay	0.00	5,041.73	0.00
Total outpatient care	2,220.40	549.89	1,235.75
Total	4,333.85	7,705.07	3,419.58

### The costs of 20-month standardized care (Models A, B and C)

We estimated that the total costs of Models A, B and C in 2020 USD from the health sector perspective were $14,781 per patient for Model A, $12, 113 per patient for Model B and $7,572 per patient for Model C “Table 9”. For Models with hospitalization (Models A and B), in-patient care accounted for over half of the cost of care and for Model C—the fully ambulatory model, out-patient care accounted for 61% of the cost of care. Drug costs, total base-line and patient follow-up costs were identical for these three models.

**Table 9:** The total cost of Models A, B and C in USD.

	Model A	Model B	Model C
Total baseline	285,60	285.60	285.60
Total follow up	1,701.12	1,701.12	1,701.12
Total drugs	954.36	954.36	954.36
Total inpatient stay†	10,209.50	6,386.19	0,00
Total outpatient care	1,630.59	2,785.52	4,631.06
Total	14,781.16	12,112.78	7,572.14

### Cost comparison of Models D, E and F with Models A, B and C

**Fig 1:**
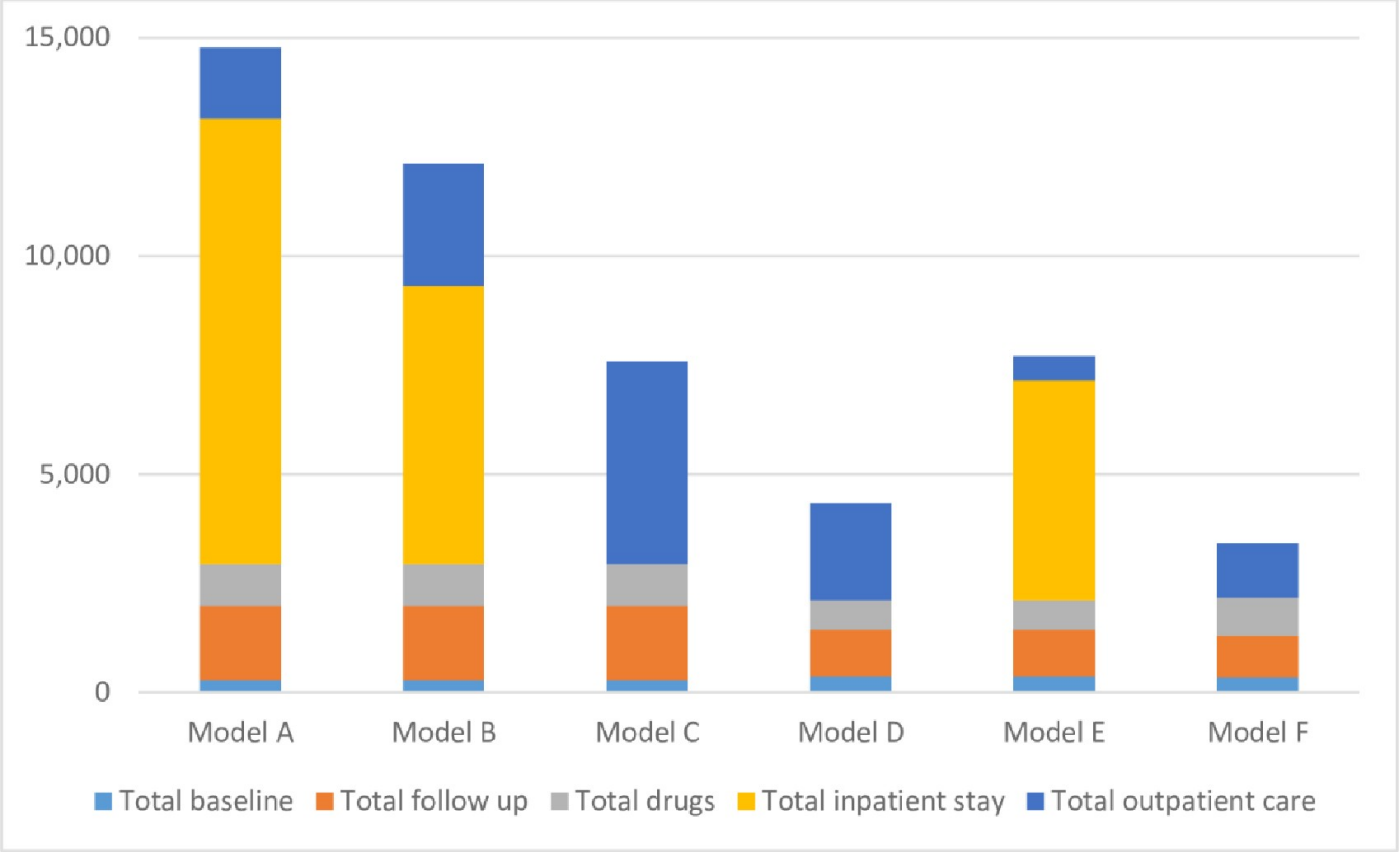
Total costs for six models of RR/MDR-TB treatment in Nigeria.

We estimated that the total costs for models A, B, C, D, E and F were $14,781, $12, 113, $7,572, $4,334, $7,705 and $3,420 respectively “Fig 1”. The mean cost for Models A, B and C was $11,489 while that for Models D and E is $6,019 resulting in a cost savings of $5,470 (48%) per patient for patients managed using Models D and E rather than A, B and C. Transitioning from Models D and E to Model F would result in further cost savings of $914 to $4,285 (21 to 56%) for every patient placed on Model F instead of Model D or E.

## Discussion

The costs of Models D, E and F are $4,334, $7,705 and $3,420 respectively, and these total costs are similar to those of shorter treatment regimen used in Ethiopia and South Africa [20]. It was however, difficult to compare cost drivers with Ethiopia and South Africa because different cost components were used to build the costs; while we built staff costs and the costs of consumables into in-patient and out-patient care; they were treated as separate cost components in the evaluation of the short treatment models in Ethiopia and south Africa [20]. As expected, Model E which differs from Model D only by the hospitalization of patients for 120 days, is almost twice the cost of Model D. Therefore, using a treatment model of nine month’s duration, with an injectable agent without hospitalization results in a cost savings of $3,371 per patient as compared to the same model with hospitalization for 120 days. However, the use of ambulatory models requires the additional cost of strong patient support structures in the community and patient and community education on infection control to minimize community transmission of RR/MDR-TB.

Model F, which is the model currently recommended by WHO [21], is the least costly of the six models. Though it contains bedaquiline which is the most expensive anti-TB drug to be used in Nigeria, the cost of bedaquiline is offset by negating the need for daily injections for 120 days and monthly audiometry for four months which are required for patients on Am or Km. Though Models D and F do not incur in-patient costs, their out-patient costs reflect four additional months of out-patient visits and home visits as compared to Model E. Outpatient costs for Models D and F at $2,220, and $1,236 are responsible for 51 and 36% of the health sector costs of RR/MDR-TB care in these models. These costs which include home-visits by several cadre of staff at the state and local government level perhaps if stream-lined, could reduce the cost of TB care substantially while still supporting patients adequately. For instance, now that programmatic management of drug-resistant TB has become ingrained in the Nigerian health system, oversight visits by the state team which is the most expensive home visit may no longer be required on a routine basis.

The costs of Models A, B and C are $14,781, $12,113 and $7,572 respectively, which are markedly lower than the health sector costs of managing RR/MDR-TB in high income countries [9,30,31] but higher than the cost of treating RR/MDR-TB in similar low and middle income countries [6]. The main drivers of the differences in cost between the 20-month models of care and the shorter treatment regimen, are the duration of treatment and whether in-patient care was provided as part of care. Models A—C require a treatment duration of at least 20 months while Models D–F require treatment for 9 months. Replacing Models A–C with Models D and E reduced the costs of RR/MDR-TB care in Nigeria by approximately 48% (assuming an equal distribution of patients between all the models). However, even with the current costs of Models D-F, Phasing out models D and E and transitioning to Model F would result in cost savings of $914 to $4,285 (21 to 56%) for every patient placed on Model F instead of Model D or E. However, it is important to note that some patients might still require in-patient care due to their clinical condition and this is not taken into account in our calculations for Model F.

In selecting a model to utilize, in addition to considering the cost of the model, it is important to consider the outcome of treatment as well as whether a particular model is feasible given the allocation of existing resources. Shorter treatment regimen have been shown to be feasible in countries similar to Nigeria–several low and middle income countries in West and Central Africa have successfully implemented standardized nine to twelve month treatment regimen for MDR-TB [18,19,32] with up to 20% of one study cohort co-infected with HIV [32]. These countries achieved treatment success of 82% to 89% [18,19,32] and there is evidence that replacing a second-line injectable agent with Bdq in a shorter treatment regimen improved treatment outcomes further [25,26]. However, these countries went to great lengths to ensure optimal patient monitoring; some of these countries relied on a structured system for daily treatment delivery which had been tried and tested when longer treatment regimens were being utilized [18]. Patient monitoring included daily visits to the MDR-TB facility for DOTS which became weekly visits during the continuation phase [19]. Even patients outside the immediate vicinity of the treatment center were supported with daily visits by regional trained agents but still had to visit the MDR-TB facility monthly, while patients with poor clinical condition were hospitalized during the intensive phase [19]. Similar support structures are currently in use in Nigeria with highly specialized State teams that provide technical support to all centers managing DR-TB patients as well as DOTS officers, TB and leprosy supervisors at local government level and DR-TB focal persons in all states who have well-delineated roles and a standardized schedule for patient monitoring. In addition, Nigeria utilizes patient supporters who are community volunteers and support patients through all aspects of their care including DOT, obtaining routine investigations and adhering to infection control measures.

Another consideration prior to adopting a shorter regimen is adequate preparation for the use of new and re-purposed drugs which are used to constitute these regimen and may cause increased complexity of the health system thus requiring large scale training of staff, improved patient monitoring and accelerated laboratory processing capacity. For example, MFX is a re-purposed drug thought to induce prolongation of the QT interval in cardiac rhythm as is the new drug Bdq [33,34]. As such, before these drugs are included in a treatment regimen, the infrastructure and trained staff for performing electrocardiographs need to be in place and the changes in the timing and frequency of monitoring investigations updated in patient monitoring plans.

Our study is a cost analysis not a cost-effectiveness study. The purpose of our study was to compare the costs of nine month treatment models with the costs of 20-month standardized care rather than comparing the effectiveness of different management strategies which should be the focus of future studies. This cost analysis is subject to a number of limitations. There has been 83.5% inflation since the time the costs of one bed day, out-patient visits and meetings were derived. Also, the cost available for LPA was for first line LPA alone, not first and second-line LPA which may result in our under-estimating the cost. We did not include the cost of managing adverse drug reactions (ADRs) which are likely to be substantial due to the sheer number of medications the patients are taking and the use of anti-retroviral medications (ARVs) by HIV co-infected patients which may result in drug interactions. This was because we calculated costs based on strict adherence to two Nigerian national guidelines, but to accurately quantify the costs of ADRs, we would need to obtain the frequencies and types of ADRs from a cohort study. We also did not capture patient costs which contribute significantly to the overall cost of managing RR/MDR-TB but focused on the health sector perspective. Despite these limitations, our study provided detailed comparative information on the health system costs of treating RR/MDR-TB with six models of care in the same geopolitical region. We showed that the least costly model to manage RR/MDR-TB in Nigeria, is Model F—the fully ambulatory, fully oral bedaquiline-containing shorter treatment regimen currently recommended by WHO.

## Conclusion

Model D and E which are models of RR/MDR-TB care in Nigeria that utilize a shorter treatment regimen of nine months’ duration with second-line injectable drugs are estimated to cost $4,334 and $7,705, respectively. Model F, which is based on a shorter, all oral, regimen currently recommended by WHO to manage patients with MDR-TB who have not been previously exposed to second-line treatment for more than a month, who do not have extensive TB disease or severe extra pulmonary TB and who do not have resistance to a fluoroquinolone is estimated to cost $3,420. Replacing Models A–C with Models D and E reduced the costs of RR/MDR-TB care in Nigeria by approximately 48%. Further, transitioning from Models D and E, the models currently in use in Nigeria to Model F, would result in further cost savings of $914 to $4,285 (21 to 56%) for every patient placed on Model F instead of Model D or E. Therefore, if the improved outcomes of patients managed using bedaquiline-containing shorter treatment regimen in other countries [25,26] can be attained in Nigeria, Model F would be the recommended choice for the scale up RR/MDR-TB care in Nigeria.

## Supporting information

S1 Table Frequencies of laboratory tests and patient monitoring visits per patient and model for Models A, B and C according to the Nigerian DR-TB guideline in 2016. (DOCX)

S1 File Cost data for Models A-F. (XLSX)
