# Correction: Cost comparison of nine-month treatment regimens with 20-month standardized care for the treatment of rifampicin-resistant/multi-drug resistant tuberculosis in Nigeria

**DOI:** 10.1371/journal.pone.0259492

**Published:** 2021-10-28

**Authors:** Florence O. Bada, Nick Blok, Evaezi Okpokoro, Saswata Dutt, Christopher Akolo, Patrick Dakum, Alash’le Abimiku

The affiliation for the fourth author is incorrect. Saswata Dutt is not affiliated with #5 but with #4:

Department of Prevention, Care and Treatment, Institute of Human Virology, Abuja, Nigeria
