# Correction: Cost comparison of nine-month treatment regimens with 20-month standardized care for the treatment of rifampicin-resistant/multi-drug resistant tuberculosis in Nigeria

**DOI:** 10.1371/journal.pone.0321797

**Published:** 2025-04-04

**Authors:** The PLOS One Staff

The affiliation for the fourth author is incorrect. Saswata Dutt is not affiliated with #4 but with #5:

Department of Prevention, Care and Treatment, Institute of Human Virology, Abuja, Nigeria.

The publisher apologizes for the error.
