# Supplementary material for: Cost comparison of nine-month treatment regimens with 20-month standardized care for the treatment of rifampicin-resistant/multi-drug resistant tuberculosis in Nigeria
Source: PLoS One. 2020 Dec 1;15(12):e0241065. doi: 10.1371/journal.pone.0241065 (PMC7707487; doi:10.1371/journal.pone.0241065)
Supplement: S1 Table — (DOCX) [file pone.0241065.s001.docx]

**S1 Table Frequencies of laboratory tests and patient monitoring visits per patient and model according to the Nigerian DR-TB guidelines for Models A, B and C**

|  |  | **Model A** | | **Model B** | | **Model C** | |
| --- | --- | --- | --- | --- | --- | --- | --- |
|  | Unit | Frequency in intensive phase | Frequency in continuation phase | Frequency in intensive phase | Frequency in continuation phase | Frequency in intensive phase | Frequency in continuation phase |
| **I. DR-TB Diagnostic tests** | | | | | | | |
| GeneXpert | Test | 1 | - | 1 | - | 1 | - |
| Sputum smear | Smear | 1 | - | 1 | - | 1 | - |
| Culture – Liquid | Test | 1 | - | 1 | - | 1 | - |
| 1^st^ and 2nd line DST - Solid culture | Test | 1 | - | 1 | - | 1 | - |
| **II. Baseline tests & routine laboratory tests** | | | | | | | |
| X-ray | Test | 2 | 2 | 2 | 2 | 2 | 2 |
| ENT consultation | Consult | 1 | - | 1 | - | 1 | - |
| Audiometry test | Test | 9 | - | 9 | - | 9 | - |
| E, U, Cr | Test | 8 | - | 8 | - | 8 | - |
| Thyroid function test | Test | 2 | 2 | 2 | 2 | 2 | 2 |
| LFT | Test | 3 | 4 | 3 | 4 | 3 | 4 |
| HIV Test | Test | 1 | - | 1 | - | 1 | - |
| Pregnancy test | Test | 0.5 | - | 0.5 | - | 0.5 | - |
| **III. Drugs** | | | | | | | |
| Pyrazinamide 400 mg | Tablet | 972 | 1,488 | 972 | 1,488 | 972 | 1,488 |
| Kanamycin 1 g vial | Vial | 243 | - | 243 | - | 243 | - |
| Levofloxacin 250 mg | Caplet | 729 | 1,116 | 729 | 1,116 | 729 | 1,116 |
| Prothionamide 250 mg | Tablet | 729 | 1,116 | 729 | 1,116 | 729 | 1,116 |
| Cycloserine 250 mg | Capsule | 729 | 1,116 | 729 | 1,116 | 729 | 1,116 |
| Pyridoxine 10 mg | Tablet | 729 | 1,116 | 729 | 1,116 | 729 | 1,116 |
| **IV. Inpatient stay** | | | | | | | |
| Inpatient hospitalization days | Bed day | 243 | - | 152 | - | - | - |
| **V. Outpatient consultations and supervision** | | | | | | | |
| Consultation at treatment center /Monthly clinic visit | Visit | - | 12 | 3 | 12 | 8 | 12 |
| Visits to collect medication at DOT center/ DOT at DOT center | Visit | - | 24 | 6 | 24 | 16 | 24 |
| Home visit - by DOT officer | Home visit | - | 24 | 91 | 24 | 243 | 24 |
| Home visit by DR TB focal person | Home visit | - | 12 | 3 | 12 | 8 | 12 |
| Home visit by TBL supervisor | Home visit | - | 12 | 3 | 12 | 8 | 12 |
| Quarterly state team meeting | Meeting | - | 4 | 1 | 4 | 3 | 4 |
| Quarterly state team home visit | Home visit | - | 4 | 2 | 4 | 2 | 4 |
| **VI. Follow-up DR-TB testing** | | | | | | | |
| Sputum smear | Smear | 8 | 12 | 8 | 12 | 8 | 12 |
| Culture - Liquid | Test | 8 | 6 | 8 | 6 | 8 | 6 |
| 1^st^ and 2nd line DST - Solid culture | Test | 0.2^ | 0 | 0.2^ | 0 | 0.2^ | 0 |

List of abbreviations: Cr=creatinine, DOT=directly observed treatment, DR=drug resistant, DST=drug susceptibility testing, E=electrolytes, ENT=ear nose throat, TB=tuberculosis, TBL=tuberculosis and leprosy, U=urea, LFT=liver function test

^ A conservative estimate of DR-TB patients who are still culture positive after four months of DR-TB treatment and thus require repeat second line DST.
